# Anticancer activity of *Astragalus* polysaccharide in human non-small cell lung cancer cells

**DOI:** 10.1186/s12935-017-0487-6

**Published:** 2017-12-04

**Authors:** Chao-Yan Wu, Yuan Ke, Yi-Fei Zeng, Ying-Wen Zhang, Hai-Jun Yu

**Affiliations:** 1 Department of Integrated Traditional Chinese Medicine and Western Medicine, Zhongnan Hospital of Wuhan University, Donghu Road 169#, Wuchang District, Wuhan, 430071 Hubei China; 2 Department of Radiation and Medical Oncology, Zhongnan Hospital of Wuhan University, Donghu Road 169#, Wuchang District, Wuhan, 430071 Hubei China

**Keywords:** *Astragalus* polysaccharide, Anticancer activity, Non-small cell lung cancer cells, NF-κB

## Abstract

**Background:**

We have reported that Chinese herbs *Astragalus* polysaccharide (APS) can inhibit nuclear factor kappaB (NF-κB) activity during the development of diabetic nephropathy in mice. NF-κB plays important roles in genesis, growth, development and metastasis of cancer. NF-κB is also involved in the development of treatment resistance in tumors. Here we investigated the antitumor activity of APS in human non-small cell lung cells (A549 and NCI-H358) and the related mechanisms of action.

**Methods:**

The dose–effect and time-effect of antitumor of APS were determined in human lung cancer cell line A549 and NCI-H358. The inhibition effect of APS on the P65 mRNA and protein was detected by reverse transcriptase-PCR (RT-PCR) and Western blot in A549 cells respectively. The inhibition effect of APS on the p50, CyclinD1 and Bcl-xL protein was detected by Western blot in A549 cells respectively. The effect of APS on NF-κB transcription activity was measured with NF-κB luciferase detection. Finally, the nude mice A549 xenograft was introduced to confirm the antitumor activity of APS in vivo.

**Results:**

Cell viability detection results indicated that APS can inhibit the proliferation of human lung cancer cell line A549 and NCI-H358 in the concentration of 20 and 40 mg/mL. NF-κB activator Phorbol 12-myristate13-acetate (PMA) can attenuate the antitumor activity of APS in both cell lines, but NF-κB inhibitor BAY 11-7082 (Bay) can enhance the effect of APS in both cell lines. In vivo APS can delay the growth of A549 xenograft in BALB/C nude mice. APS can down-regulate the expression of P65 mRNA and protein of A549 cells and decrease the expression of p50, CyclinD1 and Bcl-xL protein. The luciferase detection showed that the APS could reduce the P65 transcription activity in A549 cells. PMA can partially alleviate the inhibition activity of P65 transcription activity of APS in A549 cells, and Bay can enhance the down-regulation of the P65 transcription activity induced by APS in A549 cells.

**Conclusion:**

APS has a significant antitumor activity in human lung cancer cells A549 and NCI-H358. NF-κB inhibition may mediate the antitumor effect.

## Background

Cancer is a major public health problem worldwide [1]. Surgery, radiation, and chemotherapy are the standard cancer therapies. For advanced tumors, chemotherapy is the treatment of choice, which is mostly associated with severe adverse events. Development of new antitumor drugs with no or low toxicities is on the agenda. Traditional Chinese Medicine (TCM) and herbal medicines have been used in the treatment of cancer for 1000 of years in China, Japan, South Korea, and other Asian countries. Numerous studies have reported that Chinese herb medicines acting as adjuvant therapy can enhance the efficacy and decrease the side effects during the cancer treatment with chemo- and radio-therapy [2]. In the search for new cancer therapeutics with low toxicity and few side effects, TCM shows promise. But TCM is often considered as the alternative medicine among western scholars, and the rationale and method between TCM and Western Medicine are totally different. The reluctance of western academia on TCM is mainly on account of the lack of clinical and pre-clinical studies [3].

In China, one commonly used herb, *Astragalus membranaceus*, has a long history of use in Traditional Chinese Medicine. It is now commonly used as an immunomodulating agent in mixed herbal decoctions. The active pharmacological constituents of radix *A. membranaceus* include various polysaccharides, saponins, and flavonoids [4]. Among these, *Astragalus* polysaccharides (APS) has been most widely studied, mainly on their immunopotentiating properties, such as promoting dendritic cell maturation and acting as an effective adjuvant in vaccines [5]. Additionally, clinical studies showed that *Astragalus* polysaccharides could increase the effectiveness of platinum-based chemotherapy when combined with chemotherapy [6] and improve the quality of life in patients with advanced non-small cell lung cancer (NSCLC) [7]. Basic research indicates that *Astragalus* saponins could induce growth inhibition and apoptosis in human colon cancer cells and tumor xenografts [8]. Another study reported that total saponins of *A. membranaceus* (AST) possess potential antitumorigenic effects in human colon cancer cells and tumor xenografts through modulation of both mTOR and ERK signaling pathways [9]. But more studies indicated that the antitumor activity of APS may depend on its function of immune regulation [10, 11]. While there is a growing body of evidence suggesting the antitumor activity of APS, it is not well established whether APS can exert an effect of antitumor activity independent of its function of immune regulation or not.

NF-κB is a collective designation for a family of structurally-related and evolutionarily-conserved transcription factors that control the expression of numerous genes that play key roles in growth, apoptosis, tumorigenesis, differentiation, embryonic development, tumor metastasis and immune and inflammatory responses. Based on reports of NF-κB constitutive activation in numerous hematologic malignancies [12] and solid tumors, including breast and prostate tumors, melanoma and pancreatic cancer [13, 14], a role for NF-κB is now well established in multiple aspects of cancer development, progression, and treatment resistance [15]. Thus, NF-κB is a promising target of cancer therapy [16].

Among malignant cancers, lung cancer accounts for the highest mortality worldwide. Recent studies with animal models and cell culture systems have well established the relations between NF-κB and lung carcinogenesis, highlighting the significance of targeting the NF-κB signaling pathway for lung cancer treatment and chemoprevention [17]. Although lung tumors are histologically heterogeneous, tumor samples obtained from lung cancer patients showed high levels of NF-κB activation in both small cell lung cancer (SCLC) and NSCLC, and NF-κB activation is significantly associated with disease advancement in TNM stages and poor prognosis in lung cancer patients [18]. NF-κB is a positive mediator of cell growth and proliferation, which serves as a negative mediator of cell apoptosis. Activation of NF-κB up-regulates expression of its responsive genes in cancer cells; these genes include proliferative genes (such as Cyclins D and E), anti-apoptotic genes (such as Bcl-XL, cIAP1, cIAP2, XIAP, A20, TRAF-2 and c-FLIP) and pro-angiogenesis genes (such as VEGF). Additionally, NF-κB is constitutively activated in a variety of cancers; both chemotherapeutics and radiation induce NF-κB activation in cancer cells, thereby contributing to resistance to these therapies [19]. Thus, it is assumed that inhibition of NF-κB will increase the efficacy of anticancer therapeutics.

Inhibition of NF-κB signaling with various approaches has been shown to augment the efficacy of chemotherapeutics and radiation in killing cancer cells in vitro and in vivo [17]. We have reported that APS can inhibit the NF-κB activity during the development of diabetic nephropathy in mice [20]. Here, the present study investigated the antitumor activity of APS in human lung cancer cells A549 and NCI-H358 and whether its antitumor activity is mediated by NF-κB activity inhibition. Results showed that APS can significantly inhibit the proliferation of A549 and NCI-H358 cells and down-regulate p65 and p50 expression and NF-κB transcription activity.

## Methods

### Materials

Human non-small lung cancer cell A549 and NCI-H358 were purchased from CCTCC (China Center for Type Culture Collection, Wuhan, China). APS injection was purchased from Tianjing Cinorch Pharmaceuticals Company. The MTS reagent (3-(4,5-dimethylthiazol-2-yl)-5-(3-carboxymethoxyphenyl)-2-(4-sulfophenyl)-2*H*-tetrazolium) was purchased from Promega (Madison, WI, USA). Antibodies for p65, p50 and Bcl-xL were obtained from Santa Cruz Biotechnology (Santa Cruz, CA, USA); antibodies for β-actin were obtained from Boster Biotechnology (Wuhan, China). The pNF-κB-Luc reporter construct was obtained from BD Biosciences Clontech (Palo Alto, CA, USA). PMA (Sigma-Aldrich, MO, USA) was dissolved in DMSO and used at a final concentration of 50 ng/mL. Bay (Sigma-Aldrich, MO, USA) was dissolved in DMSO and used at a final concentration of 10 μM.

### Cell culture and cell viability assay

A549 cells and NCI-H358 were cultivated in F-12K Medium and RPMI respectively supplemented with 10% fetal calf serum, 100 units/mL of penicillin, and 100 μg/mL of streptomycin. Cells were routinely grown under a humid environment at 37 °C, 5% CO_2_, and passaged twice a week. Cell viability was analyzed using the MTS assay following the manufacturer’s protocol. Briefly, cells were plated in each well of a 96-well tissue culture plate with 100 μL of medium (3000/well). Cells became 40–50% confluent after 24 h of incubation. The medium was then replaced with 100 μL of fresh medium containing different concentrations of APS and DMSO for various periods of time with or without the 12 h pre-incubation of the NF-κB activator PMA or NF-κB inhibitor Bay. The MTS assay was performed after a designated period of time using the protocol provided by Promega. Twenty microliters of MTS solution were added to each well using a multiple tip-pipette, and cells were incubated at 37 °C for 1–2 h. The optical density of each well was then read at 490 nm. The data were expressed as a percentage of the values obtained from cells cultured under the same conditions without drug.

### Tumor growth xenograft study

Six-week-old inbred female BALB/C nude mice were obtained from Hubei Provincial Laboratory Animal Center. The study was approved by The Animal Care and Welfare Committee of Zhongnan Hospital of Wuhan University. The animals were fed and cared for according to the Guiding Principles for Care and Use of Laboratory Animals of Europe. A549 lung adenocarcinoma cells (5 × 10^6^) were subcutaneously inoculated in the upper left flank on day 1. When the diameters of the tumors were 5 mm, the mice were randomly divided into negative control, APS, and positive control (n = 8 each) groups. Mice in the negative control (NS) group were administered NS (normal saline) for 30 days through peritoneal injection. Mice in APS group were administered with 300 mg/kg per day for 30 days. Mice in the positive control group were injected intra-peritoneally with 8 mg/kg Cisplatin (DDP) once a week for 3 weeks. Tumor size was measured using calipers every 3 days, and the volume was calculated using the following formula: (L × W^2^)/2, where L equals length and W equals width. Mice were euthanized on day 30 with pentobarbital injection.

### RT-PCR and Western blot measurement

RT-PCR was used to detect the expression level of p65 mRNA. Western blot were applied to measure the expression levels of p65, p50, CyclinD1 and Bcl-xL protein.

Total RNA was isolated from A549 cells using TRIzol reagent and reverse-transcribed with the SuperScript II kit (Invitrogen) following the manufacturer’s protocol. The cDNA was then subjected to PCR amplification with the following primers: p65 forward, 5′-GGGAAGGAACGCTGTCAGAG-3′, and p65 reverse, 5′-TAGCCTCAGGGTACTCCATCA-3′, beta-actin forward, 5′**-**GCATGGAGTCCTGTGGCAT-3′, and beta-actin reverse, 5′-CTAGAAGCATTTGCGGTGG-3′. The PCR conditions and thermal cycles were as follows: initial denaturation at 94 °C for 5 min, then denature at 94 °C for 30 s, anneal at 58 °C for 60 s, extend at 72 °C for 45 s with 25 cycles, finally extension at 72 °C for 10 min. The PCR products were separated in a 1.5% agarose gel containing ethidium bromide and visualized under UV light.

Western blot analysis was performed to detect the protein expression level of p65 and Cyclin D1. In brief, cells were lysed in a buffer containing 50 mM Tris, pH 7.4, 50 mM NaCl, 0.5% NP-40, 50 mM NaF, 1 mM Na_3_VO_4_, 1 mM phenylmethylsulfonyl fluoride, 25 mg/mL leupeptin, and 25 mg/mL aprotinin. The lysates were centrifuged at 15,000*g* for 15 min and the supernatants were collected. Proteins were separated by SDS-PAGE, transferred to a PVDF membrane, and blotted with specific antibodies against proteins of interest, as described in the figure legends.

### NF-κB activity assay

The NF-κB-luciferase plasmid construct (pNF-κB-Luc, BD Biosciences Clontech, Palo Alto, CA) was used to study NF-κB activity. A549 cells (2 × 10^6^) were initially seeded in 75-mm flasks containing 5 mL of DMEM medium with supplements. Cells reached 80% confluence after 24 h of incubation and were washed once with cold PBS. Lipofectin–DNA complexes were prepared by incubating 25 μL of lipofectin reagent with 4 μg of reporter plasmid DNA in 0.2 mL of RPMI-1640 medium without supplements for 40 min at room temperature (22 °C). The lipofectin–DNA complexes were diluted in 1 mL serum-free RPMI-1640 medium, added to the cells, and incubated for 5 h at 37 °C. Four milliliters of complete RPMI-1640 medium (with 15% FBS) were then added and the cells incubated overnight. The cells were then plated into 24-well plates at a density of 100,000 per well. After 48 h of transfection, cells were treated with APS and DMSO for 6 h with or without the 6 h pre-incubation of the NF-κB activator PMA or NF-κB inhibitor Bay. The manufacturer’s protocol for assaying luciferase activity was followed with minor modifications. Cells were washed once with cold PBS in the absence of Mg^2+^ and Ca^2+^ and lysed with 100 μL of reporter lysis buffer (Promega, Madison, WI). The lysate was briefly centrifuged and cell particulates were removed. The protein concentration was determined using a kit from Pierce (Rockford, IL). Luciferase assays were performed using Turner Biosystems Modulus Microplate Multimode Reader with 30 μL of luciferase assay reagent mixed with 50 μL of protein extract. The relative light readings were normalized for the amount of protein in each sample, and the results presented as relative changes in luciferase activity.

### Statistics analysis

One-way ANOVA was used for assessing the differences among groups of data, followed by Dunnett analysis with p < 0.05 as the level of statistical significance. All statistical analyses were performed with Graphpad Prism software Version 5.0 (San Diego, CA).

## Results

### Effects of APS on the viability of A549 and NCI-H358 lung cancer cells

**Fig. 1:**
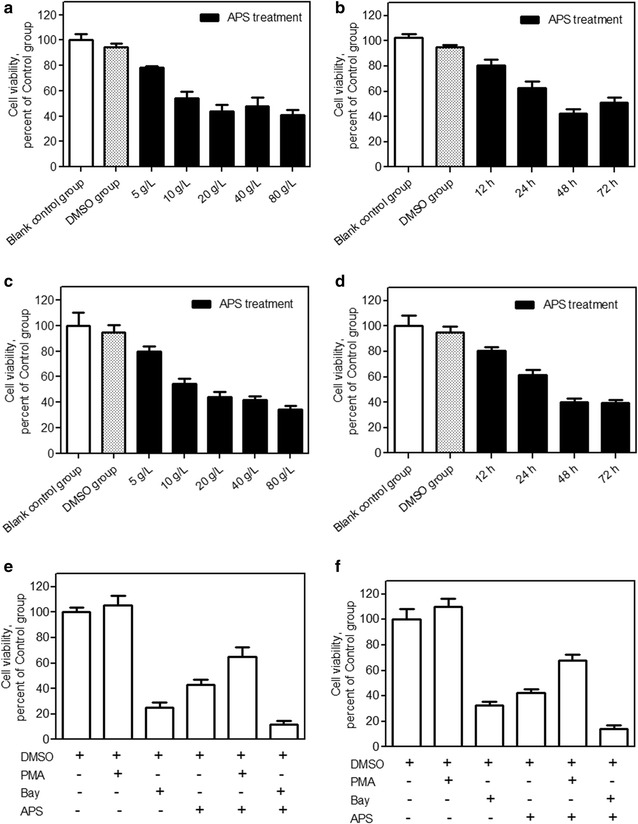
APS inhibits A549 and NCI-H358 cell growth in a concentration- and time-dependent manner. A549 cells were treated with different concentration of APS for 24 h **a** and with 20 mg/mL APS for different time **b**, NCI-H358 cells were treated with different concentration of APS for 24 h **c** and with 20 mg/mL APS for different time **d**. The growth inhibition of 20 mg/mL APS on A549 and NCI-H358 cells with or without the 12 h pre-incubation of the NF-κB activator PMA or NF-κB inhibitor Bay was shown in **e**, **f** respectively. Representative data of three independent experiments are shown

We first examined the effects of APS on the viability of A549 and NCI-H358 cells. As shown in Fig. 1a, b, APS can inhibit A549 cell growth in a concentration- and time-dependent manner. APS at 5 mg/mL showed minor toxicity, and at 80 mg/mL reduced cell viability by around 50%. The toxicity of APS at concentrations of 20, 40, and 80 mg/mL showed no difference. Time-course studies revealed the peak toxicity of 20 mg/mL of APS after 48 h incubation. Figure 1c, d showed that APS has the same inhibition effect of viability in NCI-H358 cells. To investigate the role of NF-κB activity in the antitumor activity of APS in two cell lines, we introduced the NF-κB activator PMA or NF-κB inhibitor Bay. Compared with the APS group, NF-κB activator PMA can attenuate the proliferation inhibition of APS in two cell lines, and NF-κB inhibitor Bay can enhance the proliferation inhibition of APS in two cell lines (Fig. 1e, f).

### Effects of APS on A549 xenograft growth

**Fig. 2:**
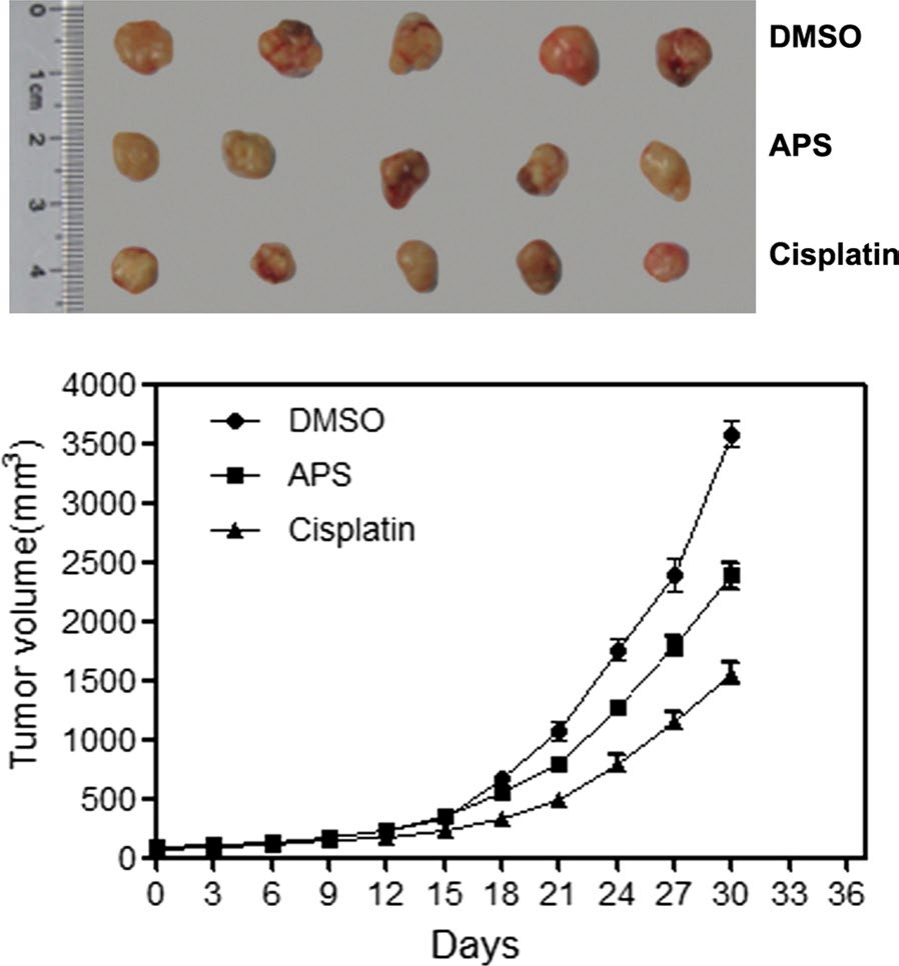
APS delays the growth of A549 xenograft in BALB/C nude mice. A549 lung adenocarcinoma cells (5 × 10^6^) were subcutaneously inoculated in the upper left flank of mice, the mice were randomly divided into three group (n = 8 each): Negative control (treated with normal saline), APS (300 mg/kg per day for 30 days through i.p injection), and positive control (8 mg/kg DDP once a week for 3 weeks). Tumor size was recorded every 3 days and the volume was calculated using the following formula: (L × W^2^)/2, where L equals length and W equals width

We further investigated the inhibitory effect of APS on the tumor growth of A549 cells in vivo. DDP was introduced as the positive control (Fig. 2). Two-way ANOVA analysis indicated that administration of APS and DDP to A549 bearing mice resulted in remarkably delayed tumor growth in comparison with the control group (p < 0.001). The growth inhibition effect was apparent after 18 days of treatment (DDP vs Control, p < 0.001 on day 18–30; APS vs Control, p < 0.05 on day 21, p < 0.001 on day 24–day 30). DDP treatment was associated with more potent antitumor activity (DDP vs APS, p > 0.05 on day 0–15, p < 0.05 on day 18, p < 0.001 on day 21–30), the inhibitory effect of tumor growth in the DDP group was stronger than that seen in the APS group.

### Effects of APS on the expression of p65 and p50 in A549 cells

**Fig. 3:**
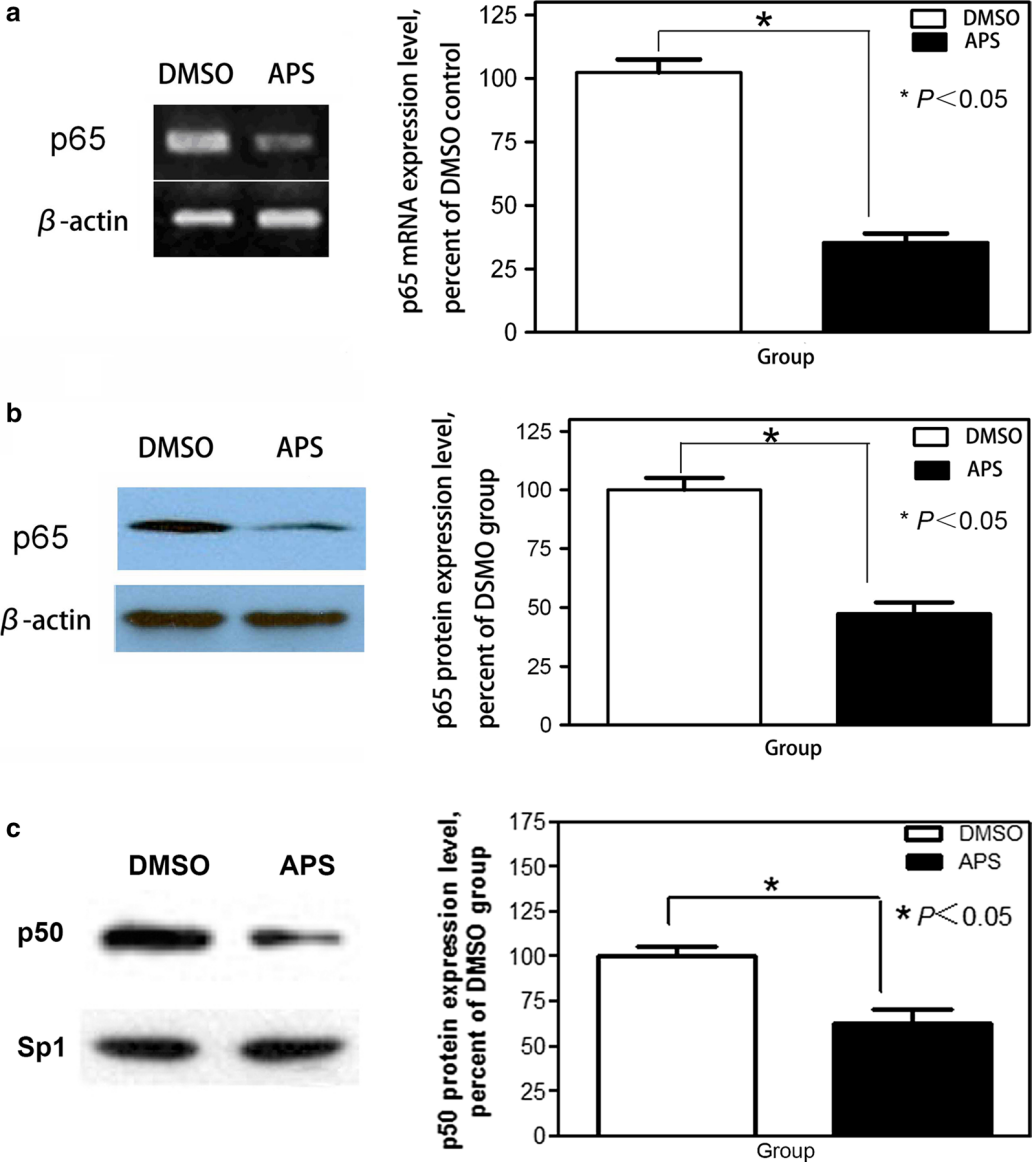
The downregulation of APS on p65 and p50 expression in A549 cells. The RT-PCR (reverse transcription PCR) and Western blot were applied to measure the expression level of p65 mRNA and protein **a**, **b**. Western blot was applied to measure the expression level of p50 protein in nuclei **c**. Representative data of three independent experiments are shown

We have reported that APS can down-regulate the expression of p65 mRNA and protein during the development of diabetic nephropathy in mice. It has not been reported whether APS can inhibit the expression of p65 mRNA and protein in human tumor cells. Here we examined the expression of p65 mRNA and protein in A549 cells treated with APS. Reverse transcription-PCR assay (Fig. 3a) revealed that p65 mRNA was decreased in A549 cells treated with APS for 24 h (APS group vs Control group, 35.87% vs 100%, *p* < 0.05). Western blot analysis (Fig. 3b) demonstrated that APS can reduce the expression of p65 in A549 cells after 48 h incubation (APS group vs Control group, 49.10% vs 100%, *p* < 0.05). We further detected the p50 expression level in the nuclei of A549 cells treated with 20 mg/mL APS. Western blot analysis (Fig. 3c) indicated that APS can decrease the expression level of p50 protein in the nuclei of A549 cells (APS group vs Control group, 62.30% vs 100%, *p* < 0.05). These data show that APS can inhibit the expression levels of p65 and p50 in human cancer cells.

### Effects of APS on the activity of NF-κB in A549 cells

**Fig. 4:**
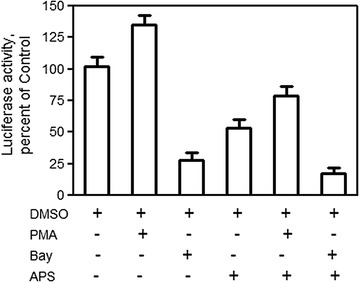
The inhibtion of APS on NF-κB transcription activity in A549 cells. The NF-κB-luciferase plasmid construct (pNF-κB-Luc) was used to study NF-κB activity. pNF-κB-Luc were transfected into the A549 cells, the cells then were treated with APS and DNSO for 6 h. The luciferase activity of NF-κB was determined according the protocol. Representative data of three independent experiments are shown

We first showed that APS can decrease the expression of p65 mRNA and protein in A549 cells. Next, we determined whether APS can down-regulate the activity of NF-κB in A549 cells (Fig. 4). We introduced NF-κB-luciferase plasmid construct to detect the effect of APS on the activity of NF-κB. Luciferase activity detection showed that APS at the concentration of 20 mg/mL can decrease NF-κB activity after 6 h of treatment (APS group vs Control group, 53.19% vs 100%, *p* < 0.05). Six hours pre-incubation with NF-κB activator PMA or NF-κB inhibitor Bay, PMA can alleviate the inhibition effect of luciferase activity of APS in A549 cell line, and Bay had a synergistic inhibition effect of luciferase activity with APS in A549 cell line. These data are consistent with the result of APS on the expression of p65 mRNA and protein in A549 cells, which showed that NF-κB activity plays an important role in the antitumor activity of APS in human lung cancer cells lines.

### Effects of APS on the expression of CyclinD1 and Bcl-xL in A549 cells

**Fig. 5:**
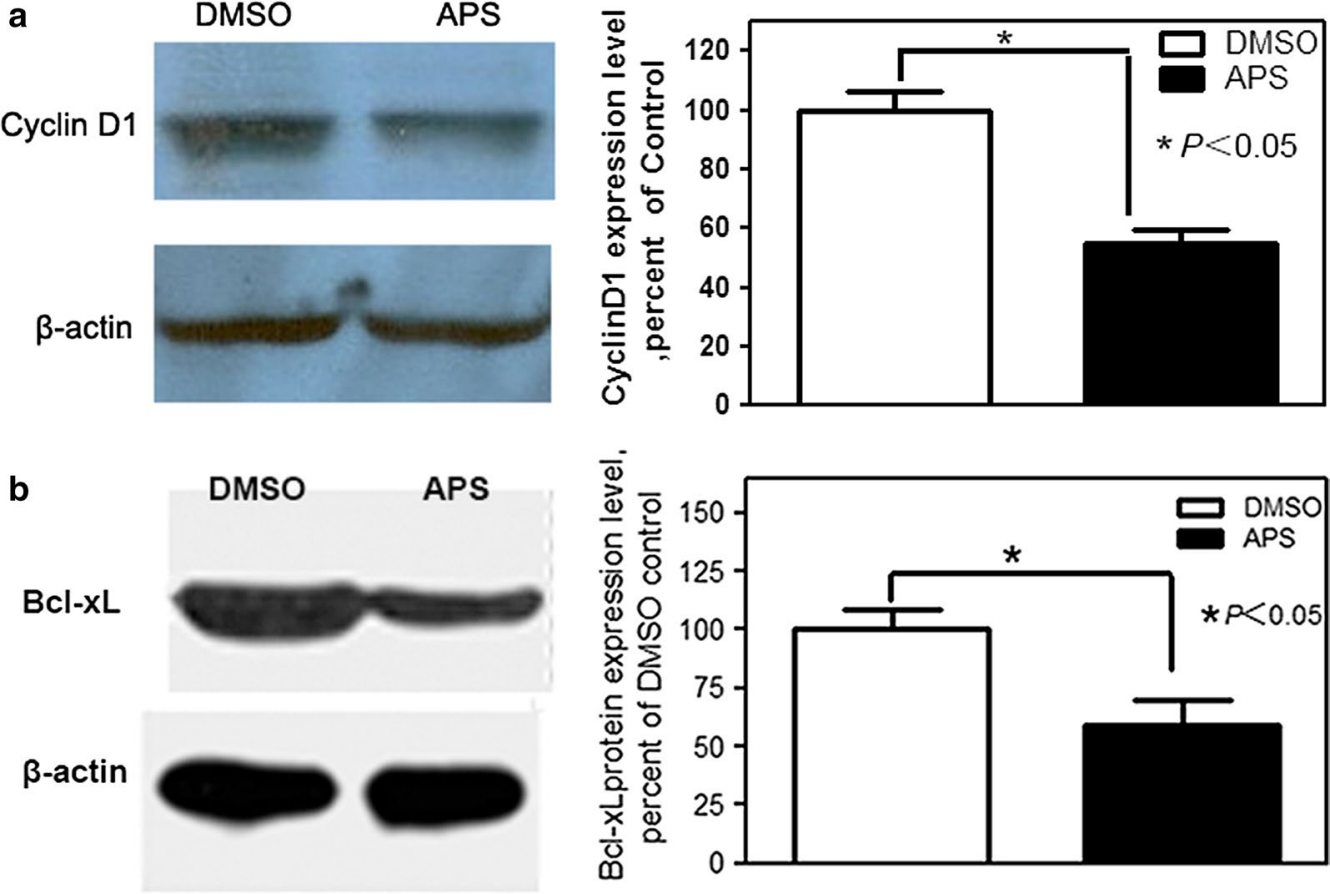
The influence of APS on CyclinD1 and Bcl-xL protein expression in A549 cells. The Western blot were applied to measure the expression level of CyclinD1 **a** and Bcl-xL **b** protein. Representative data of three independent experiments are shown

The covalently modified, activated free form of NF-κB is transported to the nucleus and binds to target DNA sequences, termed κB sites (GGGRNYYYCC), and recruits many chromatin remodeling factors to the NF-κB target promoters (Cyclin D1 and Bcl-xL), eventually leading to the activation of their target genes implicated in cell proliferation, invasion and cell death. Here we measured the effect of APS on CyclinD1 and Bcl-xL protein in A549 cells. After 72 h treatment of 20 mg/mL APS, the CyclinD1 expression level in the APS group decreased by 45.01% compared with the DMSO group (Fig. 5a, *p* < 0.05), the Bcl-xL protein expression level in the APS group reduced to 59.23% compared with DMSO group (Fig. 5b, *p* < 0.05).

## Discussion

In recent years, APS has been shown to enhance immune function in micro environments [11] and inhibit colon cancer cell growth in vivo [9]. Many studies reported that APS can improve immune functions, but it is unclear whether the antitumor activity of APS is dependent or independent on immune function. Here we performed the MTS assay in human non-small cell lung cancer cells (A549 and NCI-H358) after APS treatment. Results indicate that APS can inhibit proliferation of A549 and NCI-H358 cells in vitro, reduce the expression of p65 and p50, down-regulate the transcription activity of NF-κB, and decrease the expression of CyclinD1 and Bcl-xL protein. Further studies showed that APS can delay the tumor growth of A549 xenograft in vivo. These data show the antitumor activity in human non-small cell cancer cells for the first time, confirming that APS can suppress human tumor cells in vitro, independently of the immune system.

The present study first indicated that APS has antitumor activity in human non-small lung cancer cells. We introduced the MTS method to investigate the proliferation effect of APS on human A549 and NCI-H358 cells. Data showed that APS has concentration- and time-dependent antitumor activity in A549 and NCI-H358 cells, with the antitumorigenic effects observed in the A549 xenograft. Other studies also suggest that APS can enhance the antitumor effect of chemotherapeutic agents and diminish the side effects of chemotherapy [2]. Guo et al. reported that APS injection can raise the quality of life and survival of advanced non-small cell lung cancer patients treated with vinorelbine and cisplatin [7]. In 2007, a report showed that *Astragalus* saponins can inhibit HT-29 human colon cancer cell proliferation through accumulation in S phase and G2/M arrest, and promote apoptosis in HT-29 cells through Caspase 3 activation and poly (ADP-ribose) polymerase cleavage. The following study confirmed these results [9]. Other researchers also demonstrated that *Astragalus mongholicus* injection may inhibit proliferation and induce apoptosis in human breast cancer cell lines [21]. Our study clarified for the first time the antitumor effect in human non-small cell lung cancer cells in vitro and in vivo.

The mechanism of antitumor activity of APS is multiple and complex, although several studies have demonstrated its effect. Our study showed that APS can down-regulate p65 and p50 expression, and decrease the transcription activity of NF-κB. We also found that NF-κB activator PMA can attenuate the antitumor activity of APS in human lung cancer cell lines, NF-κB inhibitor Bay can enhance the antitumor activity of APS in the two cell lines. NF-κB is a key transcription factor involved in several pathways in cancer, which is constitutively active in most cancers. The role of NF-κB in the carcinogenesis, development, proliferation, and treatment resistance has been confirmed [22]. Thus, the constitutive activation of NF-κB has emerged as one of the most attractive targets for intervention and treatment of cancer. We have reported that APS can inhibit the NF-κB activity during the development of diabetic nephropathy in mice [20]. Here we found that APS can decrease NF-κB activity in human non-small cell lung cancer cells for the first time. Other studies also indicated that APS can prevent tumor growth through down-regulation of the expression of Akt phosphorylation in human breast cancer cells [21], modulation of both mTOR and ERK signaling pathways [9] or Caspase 3 activation [8] in human colon cancer cells.

It was previously reported that NF-κB stimulates transcription of CyclinD1, a key factor involved in G1 checkpoint control and regulator of the cell cycle and apoptotic processes [23]. Hence we suspected that prevention of NF-κB entry into the nucleus might have prevented the activation of the CyclinD1 promoter, which is responsible for active proliferation of lung cancer cells. After 72 h of treatment with 20 mg/mL APS, the CyclinD1 expression level in the APS group decreased by 45.01% compared with the DMSO group. NF-κB also can stimulate transcription of Bcl-xL, which acts as an anti-apoptotic protein by preventing the release of mitochondrial contents such as cytochrome c. Here we confirmed that the APS can reduce the protein expression level Bcl-xL in A549 cells. These data confirmed our hypothesis.

## Conclusions

In brief, our study indicates that APS can inhibit the proliferation and delay the tumor growth xenograft of human non-small lung cancer cells in vivo and in vitro through the down-regulation of NF-κB activity. We will further clarify the independent antitumor activity of APS and enrich the antitumor mechanism of APS, which will benefit the development of low toxic antitumor reagents.

## Abbreviations

APS: *Astragalus* polysaccharide
NF-κB: nuclear factor kappaB
PMA: phorbol 12-myristate13-acetate
Bay: BAY 11-7082
TCM: Traditional Chinese Medicine
NSCLC: non-small cell lung cancer
AST: *Astragalus membranaceus*
mTOR: mammalian target of rapamycin
ERK: extracellular regulated protein kinases
SCLC: small cell lung cancer
Bcl-XL: B-cell lymphoma-extra large
cIAP1 and cIAP2: cellular inhibitors of apoptosis protein 1 and 2
TRAF-2: TNF receptor-associated factor 2
c-FLIP: FLICE-like inhibitory protein
VEGF: vascular endothelial growth factor
DDP: cisplatin
XIAP: X-linked inhibitor of apoptosis protein
